# Supplementary material for: Feasibility, Safety, and Effects of an Aerobic Training Program with Blood Flow Restriction on Functional Capacity, and Symptomatology in Women with Fibromyalgia: A Pilot Study
Source: Biomedicines. 2024 Aug 19;12(8):1895. doi: 10.3390/biomedicines12081895 (PMC11351873; doi:10.3390/biomedicines12081895)
Supplement: Supplementary file 1 [file biomedicines-12-01895-s001.zip › Table S1 Abbreviations.pdf]

Table S1: Abbreviations.

ACR: American College of Rheumatology.
NSAIDs: Non-steroidal anti-inflammatory drugs.
BMI: Body mass index.
BP: Blood pressure.
RPE: Rate of Perceived Exertion.
VAS: Visual Analogue Scale.
SpO2: Oxygen saturation.
HR: Heart rate.
Kgf: kilogram force.
mmHg: Millimeter of mercury.
AE-BFR: Aerobic exercise group with blood flow restriction.
AE: Aerobic exercise group.
N: Newton.

Functional capacity

- **6MWT:** Six-minute Walk Test.
- **ISWT:** Incremental Shuttle Walk Test.
- **TUGT**: Timed Up-and-Go Test.
- **KES:** Knee Extension Strength.
- **CST**: Chair Stand Test.

Symptomatology

- **WPI:** Widespread Pain Index.
- **FIQ:** Fibromyalgia Impact Questionnaire.
- **MFI:** Multidimensional Fatigue Inventory.
- **Sscore:** Symptom Severity Score.

Hematological and biochemical parameters

- **Chol:** Total cholesterol.
- **HDL:** High-density lipoproteins.
- **LDL:** Low-density lipoprotein.
- **TG:** Triglycerides.
- **TAC:** Total antioxidant capacity.
- **MDA:** Malondialdehyde.
- **CK:** Creatine kinase
- **GGT:** Gamma-glutamyl transferase.
- **GPT:** Glutamate-pyruvate transaminase.
- **GOT:** Glutamic-oxaloacetic transaminase
- **ALP:** Alkaline phosphatase.
- **Cr:** Creatinine.
- **Uric ac:** Uric acid.
- **CoQ10:** Total coenzyme Q10.
    - **Ratio:**
        - **CoQ10/HDL**
        - **CoQ10/LDL**
        - **CoQ10/Chol**
        - **CoQ10/TG**
